# Redesigned TetR-Aptamer System To Control Gene Expression in Plasmodium falciparum

**DOI:** 10.1128/mSphere.00457-20

**Published:** 2020-08-12

**Authors:** Krithika Rajaram, Hans B. Liu, Sean T. Prigge

**Affiliations:** a Department of Molecular Microbiology and Immunology, Johns Hopkins Bloomberg School of Public Health, Baltimore, Maryland, USA; The Hebrew University of Jerusalem

**Keywords:** malaria, protein knockdown, tetracycline, tetracycline repressor, RNA aptamers, TetR-DOZI

## Abstract

One of the most powerful approaches to understanding gene function involves turning genes on and off at will and measuring the impact at the cellular or organismal level. This particularly applies to the cohort of essential genes where traditional gene knockouts are inviable. In Plasmodium falciparum, conditional control of gene expression has been achieved by using multicomponent systems in which individual modules interact with each other to regulate DNA recombination, transcription, or posttranscriptional processes. The recently devised TetR-DOZI aptamer system relies on the ligand-regulatable interaction of a protein module with synthetic RNA aptamers to control the translation of a target gene. This technique has been successfully employed to study essential genes in P. falciparum and involves the insertion of several aptamer copies into the 3′ untranslated regions (UTRs), which provide control over mRNA fate. However, aptamer repeats are prone to recombination and one or more copies can be lost from the system, resulting in a loss of control over target gene expression. We rectified this issue by redesigning the aptamer array to minimize recombination while preserving the control elements. As proof of concept, we compared the original and modified arrays for their ability to knock down the levels of a putative essential apicoplast protein (PF3D7_0815700) and demonstrated that the modified array is highly stable and efficient. This redesign will enhance the utility of a tool that is quickly becoming a favored strategy for genetic studies in P. falciparum.

**IMPORTANCE** Malaria elimination efforts have been repeatedly hindered by the evolution and spread of multidrug-resistant strains of Plasmodium falciparum. The absence of a commercially available vaccine emphasizes the need for a better understanding of *Plasmodium* biology in order to further translational research. This has been partly facilitated by targeted gene deletion strategies for the functional analysis of parasite genes. However, genes that are essential for parasite replication in erythrocytes are refractory to such methods, and require conditional knockdown or knockout approaches to dissect their function. One such approach is the TetR-DOZI system that employs multiple synthetic aptamers in the untranslated regions of target genes to control their expression in a tetracycline-dependent manner. Maintaining modified parasites with intact aptamer copies has been challenging since these repeats can be lost by recombination. By interspacing the aptamers with unique sequences, we created a stable genetic system that remains effective at controlling target gene expression.

## Abstract (precis)

Malaria elimination efforts have been repeatedly hindered by the evolution and spread of multidrug-resistant strains of Plasmodium falciparum. The absence of a commercially available vaccine emphasizes the need for a better understanding of *Plasmodium* biology in order to further translational research. This has been partly facilitated by targeted gene deletion strategies for the functional analysis of parasite genes. However, genes that are essential for parasite replication in erythrocytes are refractory to such methods, and require conditional knockdown or knockout approaches to dissect their function. One such approach is the TetR-DOZI system that employs multiple synthetic aptamers in the untranslated regions of target genes to control their expression in a tetracycline-dependent manner. Maintaining modified parasites with intact aptamer copies has been challenging since these repeats can be lost by recombination. By interspacing the aptamers with unique sequences, we created a stable genetic system that remains effective at controlling target gene expression.

## INTRODUCTION

Significant strides have been made toward reducing the global burden of malaria and associated mortality since the turn of the millennium. However, progress toward eradication has slowed down in the last few years and could be further undermined by the emergence of resistant strains of Plasmodium falciparum to many frontline antimalarials (1). Understanding the genetic and mechanistic basis of resistance, along with the identification of novel drug targets, is therefore vital for the advancement of antimalarial therapies. These efforts have been aided by a considerable improvement in annotation of the P. falciparum genome since its release in 2002, with known or putative functions assigned to over 65% of predicted genes (2). Several technological advances in recent years have changed the landscape of molecular genetics in P. falciparum from relative intractability to the experimental validation of almost a quarter of the total number of protein-coding genes. Many of these studies have yielded unexpected results. For instance, several seemingly promising drug targets were found to be completely dispensable during the symptomatic blood stage of the parasite life cycle (3–5). There are also indications that many functionally annotated proteins have acquired additional or alternative roles in *Plasmodium* (6–11). These examples highlight a continuing need for the characterization of *Plasmodium* genes in their native context, along with the improvement and expansion of our toolkit of genetic techniques.

Gene deletion studies have been greatly facilitated by the introduction of CRISPR/Cas9 to P. falciparum. However, knockouts are applicable only to the cohort of genes whose products are either dispensable during intraerythrocytic development or can be chemically or genetically bypassed (12–15). To address this, several conditional gene expression strategies have been developed that can be integrated with CRISPR/Cas9 for the functional analysis of essential blood-stage genes.

Nearly all conditional systems in *Plasmodium* involve the binding of small molecule ligands to nucleic acid/protein modules to control gene expression or protein function. Inducible FKBP-FRB dimerization by rapamycin and its analogs (rapalogs) forms the basis for two such techniques: (i) conditional DiCre recombinase-mediated excision of target genes that are flanked by loxP sites, and (ii) conditional mislocalization of FKBP fusion proteins, known as knock sideways (KS) (16–18). Destabilizing FKBP (ddFKBP) or hDHFR (human dihydrofolate reductase) domains have been used to confer ligand-dependent stability to *Plasmodium* proteins (19–21). In the absence of ligand, these fusion proteins are degraded by the proteasome. N-terminal fusions of recently developed FKBP variants can traffic proteins to the lumen of the apicoplast organelle in a rapalog-controllable fashion (22). These tools vary in dynamic range and their successful application to a target gene may be influenced by gene expression levels, subcellular protein location, or tolerance of the protein to N- or C-terminal modifications.

To overcome constraints imposed by protein structure and location, methods have been developed to control gene expression at the posttranscriptional level in P. falciparum, either by inducible degradation or sequestration of mRNA. The self-cleaving GlmS ribozyme has been successfully appended to the 3′ UTR of target genes and the resulting transcripts are cleaved in a glucosamine-dependent manner (23). Various tetracycline repressor protein (TetR)-based systems have been tested in P. falciparum (24, 25), but perhaps the most successful strategy involves the regulatable interaction of a TetR-DOZI fusion protein with a synthetic RNA aptamer array (26). TetR normally binds to its cognate double-stranded DNA (dsDNA) operator sequences to block the transcription of a target gene. This repression can be reversed by the allosteric interaction of tetracycline (TC) or its analogs with TetR. To adapt the TetR system to posttranscriptional regulation, Belmont and Niles (2010) conducted a screen for RNA aptamers that bind to TetR in a TC-regulatable fashion (27). Candidate aptamers that fulfilled these parameters were predicted to form stem-loop structures with conserved sequence motifs in the loops. Placing an RNA aptamer in the 5′ UTR of reporter genes resulted in a TC-dependent, 3- to 5-fold change in expression levels. To increase the dynamic range of the TetR-aptamer system, Ganesan et al. (2016) exploited the native translational regulation capabilities of *Plasmodium* (26). DOZI (“development of zygote inhibited”) is a *Plasmodium* RNA helicase that represses translation by assembling specific transcripts into inactive messenger ribonucleoprotein particles (mRNPs) (28). The integration of DOZI with the TetR-aptamers afforded much greater control over reporter gene expression, especially when the 5′ UTR aptamer was combined with multiple aptamer copies in the 3′ UTR (20- to 300-fold changes in induction) (26).

Since it can be challenging to simultaneously modify the 5′ and 3′ UTRs of endogenous genes, several groups have effectively utilized TetR-DOZI with an aptamer array inserted only into the 3′ UTR (5, 26, 29–44). The 3′ UTR array consists of 10 aptamer copies, separated by spacers that are identical in sequence and length. Such tandem repeats are inherently unstable and tend to be deleted by recombination. We and others have observed truncations in the aptamer array with a consequent loss of control over gene expression, thereby limiting the utility of this knockdown strategy (30, 31, 36, 37, 43). To rectify this, we modified the array to contain unique spacers while retaining the secondary structure of the aptamers. We validated the redesigned aptamer array by targeting a putative apicoplast ubiquitin-like protein (PF3D7_0815700), referred to as PfAUBL. We found that the new array does not lose its aptamers during plasmid propagation in E. coli or upon insertion into the P. falciparum genome. The redesign does not interfere with aptamer function, as demonstrated by efficient TC-dependent knockdown of the PfAUBL protein that subsequently results in parasite death, indicating an essential role for PfAUBL. The enhanced stability of the aptamer array will expand the usage of TetR-DOZI for gene function studies in P. falciparum.

## RESULTS

### Aptamer arrays undergo truncation in E. coli and in P. falciparum.

**FIG 1:**
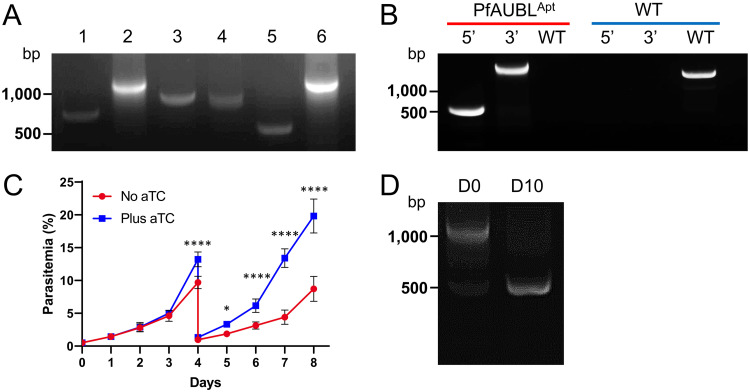
Aptamer arrays are unstable during propagation in E. coli and P. falciparum. (A) PCR amplification of the aptamer array from six bacterial colonies transformed with pMG74^PfAUBL^ plasmid. PCR products of full-length arrays are ∼1 kb in size. Smaller products in some lanes signify truncated arrays. (B) Integration of pMG74^PfAUBL^ plasmid into the 3′ UTR of the PfAUBL gene was confirmed by PCR amplification of the 5′ and 3′ loci. PCR for the unmodified locus failed to detect any residual wild-type parasites in the PfAUBL^Apt^ population (red); wild-type parasites served as a control (blue). (C) Growth of PfAUBL^Apt^ parasites in the presence or absence of aTC was monitored by daily blood smears for 8 days. The parasites were cut 1:10 on day 4. Removal of aTC caused moderate growth inhibition of PfAUBL^Apt^ parasites beginning on day 4 (two‐way ANOVA, followed by Bonferroni’s correction; ****, *P < *0.0001; **, *P < *0.01; *, *P < *0.05). Error bars represent the standard error of the mean from two independent experiments, each conducted in quadruplicate. (D) PCR amplification of the aptamer array from the aTC-treated PfAUBL^Apt^ population shows 1 kb (full-length) and 0.5 kb products, indicating that some parasites have lost a few aptamers from their array (D0). Surviving parasites at 10 days post aTC removal (D10) have a predominant 0.5 kb band, consistent with a truncated array.

The TetR-DOZI aptamer system has been successfully used for the conditional knockdown of several P. falciparum proteins. We implemented this approach to study a putative apicoplast protein called PfAUBL (PF3D7_0815700). PfAUBL was identified as essential for blood-stage survival in the genome-wide *piggyBac* mutagenesis screen conducted in P. falciparum (45). The protein contains an N-terminal apicoplast transit peptide and a ubiquitin-like domain (46). We utilized a previously published plasmid pMG74 for the insertion of a 10× aptamer array downstream of the PfAUBL coding region (26). Plasmids from individual bacterial colonies were analyzed by PCR to verify the size of the aptamer array. While some plasmids contained a full-length (∼1 kb) array, other plasmids appeared to have lost a few aptamers from their arrays (Fig. 1A). We selected a plasmid with an intact sequence-confirmed array to generate PfAUBL^Apt^ parasites. Correct insertion of the aptamer array at the 3′ UTR of PfAUBL^Apt^ was confirmed by PCR (Fig. 1B, Fig. S1 and S2A in the supplemental material).

10.1128/mSphere.00457-20.2 FIG S1 Schematic of aptamer insertion into the 3′ UTR of PfAUBL. (A) The pMG74^PfAUBL^ or pKD^PfAUBL^ plasmids were linearized by digestion with EcoRV. Homologous regions between the linearized vector and the WT PfAUBL locus are indicated by dotted lines. The promoter that drives TetR-DOZI expression is depicted by the green arrow. Positions of primers used for diagnostic PCR are indicated by blue and red arrows. “TAG” refers to C-terminal epitope tags. Abbreviations: HA1, homology arm 1; HA2, homology arm 2; UTR, untranslated region; WT, wild type. (B) Primer pairs for verifying integration of the pMG74^PfAUBL^ or pKD^PfAUBL^ plasmids into the 3′ UTR of PfAUBL are listed along with the expected sizes of the PCR products. Download FIG S1, TIF file, 2.5 MB. Copyright © 2020 Rajaram et al. 2020 Rajaram et al. This content is distributed under the terms of the Creative Commons Attribution 4.0 International license.

10.1128/mSphere.00457-20.3 FIG S2 Genotypic and phenotypic analysis of PfAUBL^Apt^ parasites from a duplicate transfection. (A) Integration of pMG74^PfAUBL^ plasmid into the 3′ UTR of the PfAUBL gene was confirmed by PCR amplification of the 5′ and 3′ loci. PCR for the unmodified locus failed to detect any residual wild-type parasites in the PfAUBL^Apt^ population (red); wild-type parasites served as control (blue). (B) PfAUBL^Apt^ parasites were seeded at 0.5% parasitemia in the presence or absence of aTC, and their growth was monitored by daily blood smears for 8 days. Parasites were cut 1:10 on day 4. Removal of aTC caused significant growth inhibition of PfAUBL^Apt^ parasites beginning on day 6 (two‐way ANOVA, followed by Bonferroni’s correction; ****, *P < *0.0001; **, *P < *0.01). Error bars represent the standard error of the mean from two independent experiments, each conducted in quadruplicate. (C) PCR amplification of the aptamer array from the aTC-treated PfAUBL^Apt^ population shows 1 kb (full-length) and smaller products, indicating that some parasites have lost a few aptamers from their array (D0). Surviving parasites at 10 days post aTC removal have a predominant smaller band, consistent with a truncated array (D10). Download FIG S2, TIF file, 1.0 MB. Copyright © 2020 Rajaram et al. 2020 Rajaram et al. This content is distributed under the terms of the Creative Commons Attribution 4.0 International license.

Polyclonal parasite populations from two independent transfections were tested for growth in the presence and absence of anhydrotetracycline (aTC), a tetracycline analog. Both parasite lines exhibited moderate growth inhibition 4 to 5 days after the removal of aTC, but continued to multiply over the course of the growth assay (Fig. 1C and Fig. S2B). To determine if the parasites replicating without aTC still contained all 10 aptamers, we performed PCR to determine the size of the array before and after aTC removal. An expected ∼1-kb band was the predominant product from aTC-treated parasites (Fig. 1D and Fig. S2C). However, one or more smaller bands were also observed, which indicated that both transfections yielded a mixed population of parasites containing various sizes of aptamer arrays. Post aTC removal, only the smaller PCR products corresponding to truncated arrays could be amplified from the surviving parasites. This suggests that PfAUBL is an essential protein and that parasites with a fully functional aptamer array died upon withdrawal of aTC. Presumably, parasites with arrays that contained fewer aptamers produced enough PfAUBL to survive in the absence of aTC.

**FIG 2:**
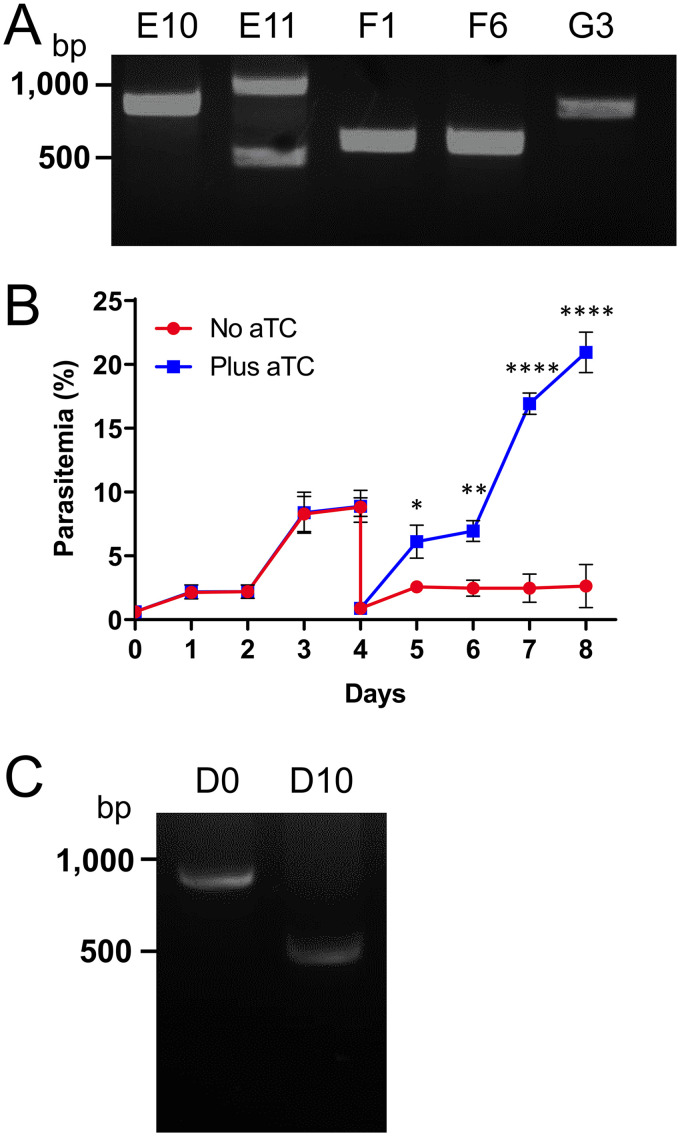
Aptamers of a PfAUBL^Apt^ clone are rapidly lost under nonpermissive growth conditions. (A) PCR amplification of aptamer arrays from cloned parasite populations reveals various array sizes. (B) Growth of clone E10 parasites in the presence or absence of aTC was monitored by flow cytometry for 8 days. The parasites were cut 1:10 on day 4. Removal of aTC caused significant growth inhibition of parasites beginning on day 5 (two‐way ANOVA, followed by Bonferroni’s correction; ****, *P < *0.0001; **, *P < *0.01; *, *P < *0.05). Error bars represent the standard error of the mean from two independent experiments, each conducted in quadruplicate. (C) PCR amplification of the aptamer array from aTC-treated clone E10 parasites (D0) shows the expected 0.8 kb product, corresponding to 8 aptamers. Parasites surviving after 10 days of no aTC treatment (D10) have a predominant 0.5 kb band, indicating that more aptamers have been lost from the original 8-aptamer array found in clone E10.

The extended time interval between parasite transfection and phenotypic analysis of transgenic lines could enable the gradual accumulation of parasites with recombined aptamer arrays. We therefore attempted to isolate a clonal parasite population with an intact array from one of the transfections. PCR analysis of five putative clones revealed aptamer arrays of various sizes. Parasites from well E11 were found to be nonclonal, and none of the remaining samples contained full-length arrays (Fig. 2A). Parasites from clone E10 had the largest array with 8 aptamers and were therefore analyzed further. Clone E10 parasites grown in the absence of aTC exhibited severe growth inhibition beginning on day 5, and this defect was much more pronounced than the previously analyzed mixed populations (Fig. 2B). However, a small proportion of aTC-depleted parasites continued to grow and replicate. When examined by PCR on day 10, the surviving parasites were found to contain a truncated array with 5 aptamers, which was confirmed by sequencing (Fig. 2C). This indicates that aptamer loss can occur within a short time frame and confers a selective advantage to parasites in nonpermissive growth conditions.

### Modification of spacers between RNA aptamers.

**FIG 3:**
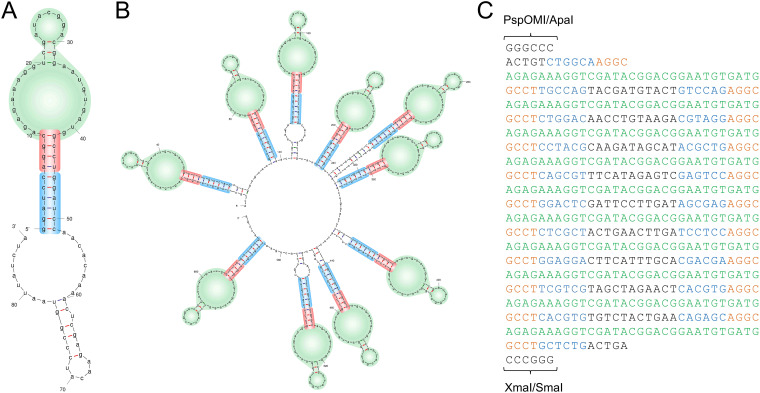
Spacers in a redesigned RNA aptamer array do not have significant secondary structure. (A) Secondary structure prediction of a single RNA aptamer from the original array using mFold. The 51-bp stem-loop structure contains a loop responsible for TetR binding (green) and a double-stranded stem (red), in part formed from residues that correspond to a BamHI site (blue). The stem-loop structure is followed by a 35-bp spacer. (B) Redesigned aptamer array which preserves the 10 stem-loop structures, but separates each aptamer with unique 11-bp spacer sequences. The BamHI sequences found in the original aptamers were replaced with unique 6-bp sequences with the same GC content (blue). (C) Sequence of the new array flanked by endonuclease sites with color coding corresponding to that used in (B). Each of the array elements now contains 23 bp of unique sequence corresponding to the spacer region (black) and part of the double-stranded stem (blue).

The TetR-aptamer array contains 10 copies of an aptamer separated by identical spacers (86 bp each and 860 bp in total). Such repetitive sequences are prone to recombination events that can lead to truncations. To minimize recombination between individual aptamers and spacers, we made the following modifications to the array. The original aptamers are 51 bp in length and are predicted to fold into stem-loop structures (Fig. 3A). Bases 1 to 10 and 42 to 51 are palindromic and can pair with each other to form a stem with a predicted melting temperature of ∼33°C. Bases 1 to 6 and 45 to 51 also encode BamHI restriction enzyme sites (Fig. 3A). To facilitate future cloning reactions that may require BamHI, we altered these bases while maintaining the same GC content (and therefore a similar melting temperature). Each modified aptamer now contained a unique palindromic 6-bp sequence on either end. The loop regions containing conserved sequence motifs that are known to be important for TetR binding were left unaltered (27). The aptamers in the original array were separated by identical 35-bp spacers. We reduced the spacer length to 11 bp and altered the sequence such that each spacer was unique and formed no significant secondary structure with the 10 stem-loop aptamers (Fig. 3B). Thus, each of the array elements now contains 23 bp of unique sequence corresponding to the spacer region (11 bp) and part of the double-stranded stem (12 bp). The redesigned spacers have higher GC content than the original spacer (52% versus 44%), thus facilitating the design of unique primers for sequencing or for future genetic manipulations. They also do not contain any common restriction endonuclease sites that could interfere with downstream cloning. We replaced the original 860-bp array in the pMG74 plasmid with the redesigned 619-bp array (Fig. 3C) to generate the pKD vector.

### Redesigned aptamer array exhibits enhanced stability in E. coli and P. falciparum.

**FIG 4:**
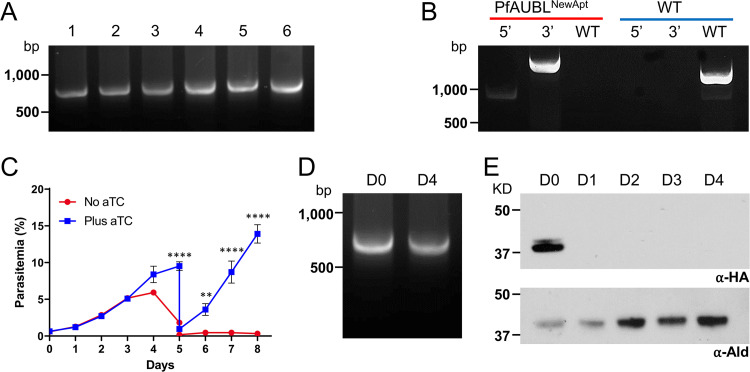
The redesigned aptamer array is stably maintained and retains responsiveness to aTC. (A) PCR amplification of the aptamer array from six bacterial colonies transformed with the pKD^PfAUBL^ plasmid shows that all colonies contain full-length (∼0.7 kb) arrays. (B) Integration of pKD^PfAUBL^ plasmid into the 3′ UTR of the PfAUBL gene was confirmed by PCR amplification of the 5′ and 3′ loci. PCR for the unmodified locus failed to detect any residual wild-type parasites in the PfAUBL^NewApt^ population (red); wild-type parasites served as a control (blue). (C) Growth of PfAUBL^NewApt^ parasites in the presence or absence of aTC was monitored by flow cytometry for 8 days. The parasites were cut 1:10 on day 5. Removal of aTC caused significant growth inhibition of PfAUBL^NewApt^ parasites beginning on day 5 (two‐way ANOVA, followed by Bonferroni’s correction; ****, *P < *0.0001; **, *P < *0.01; *, *P < *0.05). Error bars represent the standard error of the mean from two independent experiments, each conducted in quadruplicate. (D) PCR amplification of the aptamer array from aTC-treated PfAUBL^NewApt^ parasites resulted in a single band of 0.7 kb corresponding to the full-length array (D0). DNA from dying or dead parasites at 4 days post aTC removal (D4) also generated a 0.7 kb PCR product. (E) Western blotting using anti-HA antibody to probe for PfAUBL protein shows a rapid decrease in protein levels upon aTC removal. Anti-aldolase antibody was used as a loading control.

pKD plasmids with PfAUBL homology arms were isolated from a few bacterial colonies and their aptamer arrays were examined by PCR. All tested plasmids were found to contain an intact array (∼0.7 kb) (Fig. 4A). Parasites were transfected as before with a sequence-confirmed pKD plasmid to generate PfAUBL^NewApt^ parasites. The insertion of the new aptamer array at the 3′ UTR of PfAUBL was confirmed by PCR (Fig. 4B and Fig. S1). To determine if the aptamers in the redesigned array retained responsiveness to aTC, PfAUBL^NewApt^ parasites were subjected to a growth assay in the presence or absence of aTC. Growth was completely abrogated 4 days after the withdrawal of aTC, and parasites were undetectable in cultures beyond day 5 (Fig. 4C). The flow cytometric results were confirmed by manual inspection of blood smears. PCR analysis of parasites 4 days post aTC removal did not indicate any loss of aptamers from the array (Fig. 4D). A lack of parasite material in these cultures after day 5 precluded us from repeating PCR analysis of the aptamer array at the end of the 8-day growth experiment. Cultures depleted of aTC were maintained for 21 additional days, but no parasite recrudescence was observed. These results implied that the use of unique spacers between identical aptamers was sufficient to prevent recombination within the array and that the changes we made to the aptamer array did not affect the functionality of the knockdown system. PfAUBL^NewApt^ parasites maintained in permissive culture conditions for several months did not exhibit any loss of aptamers.

The pMG74 plasmid was designed to append 1× HA and 1× FLAG tags onto the C terminus of the target protein. However, we failed to detect PfAUBL in PfAUBL^Apt^ parasites using anti-HA or anti-FLAG antibodies. To enhance detection capabilities, we used a 3× HA tag in the pKD plasmid. Probing for PfAUBL with anti-HA antibody demonstrated robust protein detection in aTC-treated PfAUBL^NewApt^ parasites (Fig. 4E). To determine the efficiency of protein knockdown, we examined the levels of PfAUBL in aTC-depleted parasites over a 4-day period. Protein levels were undetectable 24 h post aTC removal, indicating that the knockdown was quick and efficient.

### PfAUBL localizes to the apicoplast.

**FIG 5:**
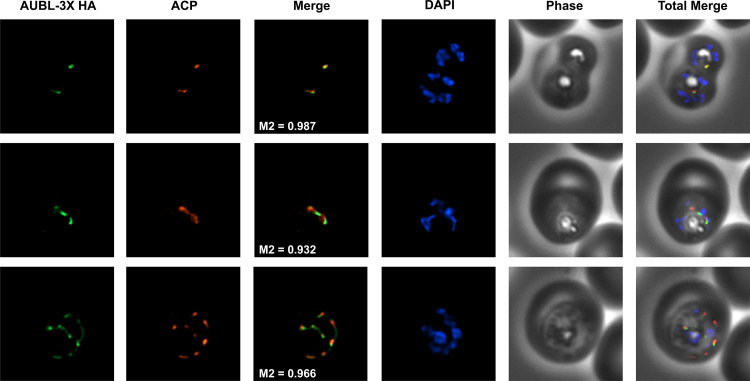
PfAUBL localizes to the apicoplast. Representative immunofluorescence microscopy images of PfAUBL^NewApt^ parasites show that PfAUBL-3× HA (green) colocalizes with the apicoplast marker ACP (red) with the following Manders’ coefficients: M1 (red in green) = 0.806, SD = 0.156, *n* = 23; and M2 (green in red) = 0.929, SD = 0.104, *n* = 23. Images represent fields that are 10 μm long by 10 μm wide.

Despite the rapid depletion of PfAUBL protein, parasites did not exhibit growth inhibition until the end of the second cycle. This is reminiscent of the delayed death phenomenon associated with drugs that interfere with essential housekeeping functions of the apicoplast (47). The predicted N-terminal bipartite signaling peptide of PfAUBL has been previously shown to direct a green fluorescent protein (GFP) fusion protein to the plastid (46). To confirm whether native full-length PfAUBL is indeed trafficked to the apicoplast, we performed immunofluorescence assays (IFA) using anti-HA antibody to determine PfAUBL localization, and observed that PfAUBL colocalizes with the acyl carrier protein (ACP), a known apicoplast marker (Fig. 5). Taken together, these results indicate that PfAUBL is an essential apicoplast protein.

## DISCUSSION

Recent years have witnessed a steady development of genetic techniques for the functional analysis of essential genes in P. falciparum. The TetR-DOZI aptamer tool for conditional gene knockdown is particularly promising because it is unhindered by structural properties or the subcellular localization of target proteins. It can also be used to control gene expression in a reversible and tunable manner by varying the dosage of the tetracycline ligand, thus permitting the evaluation of null as well as hypomorphic phenotypes. However, its broad implementation has been limited by two key issues. One is that large dynamic range control of gene expression is achieved only when aptamers are introduced on either side of the target gene’s coding region (26). While this is easy to accomplish with transgenes, modifying both the 5′ and 3′ UTRs of native genes is less straightforward. The use of a 10× aptamer array in the 3′ UTR alone has been sufficient to knock down the expression of several genes, but it may not be enough to achieve complete conditional control of genes that are expressed at high levels. Recent publications have overcome this issue by utilizing a linear vector that carries a recodonized copy of the target gene and homology arms corresponding to the 5′ and 3′ UTRs, thereby allowing simultaneous insertion of aptamers into the upstream and downstream sequences (33, 43, 44).

This study addresses a second drawback of the TetR-DOZI system, namely, the instability of the 3′ UTR aptamer array due to its repetitive nature. Our results show that truncated arrays arise frequently and that arrays below a certain threshold of aptamers are unable to exert sufficient control over gene expression. This is supported by the original study describing the TetR-RNA aptamers in which a parallel analysis of arrays containing 5 or 10 aptamers showed that the 5× array was unable to regulate reporter gene expression in yeast (26). By replacing the identical spacers in between aptamers with unique sequences, we were able to avoid array truncations. Importantly, modification of the spacers did not interfere with the functionality of the aptamers. We demonstrated this by successfully knocking down the expression of the ubiquitin-like protein PfAUBL, which led to parasite death.

The 3× HA tag appended to the C terminus of PfAUBL allowed us to monitor protein levels as well as localize it to the parasite apicoplast. Although PfAUBL expression was reduced to undetectable levels within 24 h, the parasites did not die until the end of the second cycle. This delay in growth inhibition could be due to the presence of a very small amount of PfAUBL that facilitates parasite progression into the second cycle. Alternatively, it is possible that PfAUBL belongs to a subset of essential apicoplast housekeeping proteins whose inhibition results in delayed death (47). PfAUBL has been implicated in the import of apicoplast proteins from the cytoplasm via the sERAD (symbiont ER-associated degradation) system (46). In the related apicomplexan parasite Toxoplasma gondii, knockdown of sERAD proteins blocks protein transit into the apicoplast and leads to delayed death (11, 48, 49). Further work is needed to determine if PfAUBL indeed cooperates with *Plasmodium* sERAD to support apicoplast biogenesis.

## MATERIALS AND METHODS

### Parasite culture.

P. falciparum Nf54^attB^ parasites (50) were cultured in human erythrocytes at 2% hematocrit in complete medium with Albumax (CMA) containing RPMI 1640 medium with L‐glutamine (USBiological Life Sciences), supplemented with 20 mM HEPES, 0.2% sodium bicarbonate, 12.5 μg/ml hypoxanthine, 5 g/liter Albumax II (Life Technologies) and 25 μg/ml gentamicin. Cultures were maintained at 37°C in a gas mixture containing 94% N_2_, 3% O_2_, and 3% CO_2_.

### Plasmid construction.

The pMG74 plasmid was digested with AatII and XmaI to remove 1× HA and 1× FLAG tags and the existing aptamer array (26). A small synthetic DNA fragment containing cloning sites for the aptamer array (PspOMI and XmaI) and residues encoding a 3× HA tag was inserted into pMG74 using the AatII and XmaI sites (LifeSCT). The redesigned 619-bp aptamer array (Fig. 3C) was cut with PspOMI and XmaI from a synthetic plasmid (LifeSCT) and ligated into the same sites in pMG74. The resulting plasmid was called pKD and contains the homology arm cloning sites, the 3× HA tag, and the aptamer array shown in Fig. S3A and B.

10.1128/mSphere.00457-20.4 FIG S3 pKD^PfAUBL^ plasmid annotations. (A) The relative location of various restriction enzyme sites for cloning or plasmid linearization and the sequence of the 3× HA tag in the pKD vector are shown. (B) The pKD^PfAUBL^ plasmid map shows the P. falciparum Hsp86 promoter that drives the expression of the polycistronic locus containing the TetR-DOZI fusion gene, an FRB domain with a nuclear localization signal (nFRB), and the selectable marker blasticidin deaminase (BSD). The coding regions are separated from each other by T2A skip peptide sequences. Download FIG S3, TIF file, 2.9 MB. Copyright © 2020 Rajaram et al. 2020 Rajaram et al. This content is distributed under the terms of the Creative Commons Attribution 4.0 International license.

To create PfAUBL knockdown constructs, pMG74 and pKD were digested with AscI and AatII. Homology arms HA1 and HA2 of the PfAUBL gene were amplified from P. falciparum genomic DNA using the primer pairs AUBL HA1.kd-F + AUBL HA1.kd-R and AUBL HA2.kd-F + AUBL HA2.kd-R, respectively (Table S1). The homology arms were fused in an additional PCR using the primer pair AUBL HA2.kd-F + AUBL HA1.kd-R, creating a combined HA2-HA1 fragment separated by two EcoRV sites. This fragment was inserted into digested pMG74 and pKD plasmids to generate pMG74^PfAUBL^ and pKD^PfAUBL^, respectively. The nucleotide sequence for the pKD^PfAUBL^ vector can be found in Supplemental Text S1. Prior to transfection, these plasmids were linearized with EcoRV.

10.1128/mSphere.00457-20.1 TABLE S1 Oligonucleotide primers used in this study. Download Table S1, DOCX file, 0.01 MB. Copyright © 2020 Rajaram et al. 2020 Rajaram et al. This content is distributed under the terms of the Creative Commons Attribution 4.0 International license.

10.1128/mSphere.00457-20.6 TEXT S1 Plasmid pKD sequence. Download Text S1, DOCX file, 0.01 MB. Copyright © 2020 Rajaram et al. 2020 Rajaram et al. This content is distributed under the terms of the Creative Commons Attribution 4.0 International license.

The Cas9 enzyme and the guide RNA were expressed from a modified version of pUF1-Cas9 (51). Plasmid pUF1-Cas9 (11,096 bp) was modified in several steps to accommodate the insertion of the guide RNA expression cassette found in pRS-LacZ (52). In the first step, the promoter driving Cas9 expression (Hsp86) was removed using XhoI and AflII and replaced with an adaptamer formed from 5′-phosphorylated primers pCas.XhoBtg.F and pCas.XhoBtg.R (Table S1). This plasmid was then digested with XhoI/BtgZI, blunted with T4 polymerase, and ligated. The resulting plasmid is smaller (10,221bp) and brings Cas9 expression under the control of the bidirectional calmodulin promoter. In the second step, a 340-bp region of the Bluescript plasmid backbone containing several endonuclease sites was removed through digestion with EcoRI/AatII, blunted with T4 polymerase, and ligated. In the third step, digestion with XmaI/SapI was used to remove the 3′ UTR of Cas9 (PbDHFR-TS) and a complete lac operon found in the Bluescript plasmid backbone. This region was replaced with an adaptamer formed from the 5′-phosphorylated primers pUF.NotI.F and pUF.NotI.R (Table S1) containing a NotI site for insertion of the guide RNA cassette found in pRS-LacZ. Before this insertion could take place, however, two BsaI sites in the plasmid backbone had to be removed (BsaI was to be used later to insert guide RNA sequences). The plasmid was digested with BsaI, removing a 1,992-bp region containing both recognition sites for this Type IIs endonuclease. PCR primers BsaMut.GG.F and BsaMut.GG.R (Table S1) were designed with flanking BsaI sites for Golden Gate insertion back into the plasmid, but with two single base changes to alter the original two BsaI recognition sites. Thus, after digestion of the PCR product with BsaI, and ligation back into the cut plasmid, the resulting plasmid was identical in size (8,757 bp) and contained two single nucleotide changes that removed both BsaI recognition sites. The guide RNA expression cassette from plasmid pAIO (31) was then excised with NotI and inserted into the same site bidirectionally, generating a forward and a reverse orientation. The orientation with the U6 5′ regulatory element adjacent to Cas9 was chosen with the expectation that this region would act as a 3′ UTR for Cas9 (it contains the 3′ UTR of the ribosomal L18 protein, PF3D7_1341200). Finally, the plasmid was digested with BtgZI to remove the preguide RNA and the LacZ expression cassette from pRS-LacZ (52) was excised with BsaI and inserted into this site. The final pCasG-LacZ plasmid (10,886) is smaller than the original pUF1-Cas9 plasmid and contains a guide RNA expression cassette that can be used for blue/white colony screening after insertion of a guide RNA sequence with BsaI (Fig. S4).

10.1128/mSphere.00457-20.5 FIG S4 Map of the pCasG-LacZ vector. The pCasG-LacZ plasmid schematic displays the bidirectional CAM/HOP promoter that drives the expression of the yeast dihydroorotate dehydrogenase (yDHOD) resistance gene on one side, and the Cas9 enzyme and the LacZ cassette on the other side. The LacZ cassette is flanked by the type IIS restriction enzyme site BsaI. Digestion with BsaI removes the LacZ cassette and facilitates insertion of a guide RNA (gRNA) adaptamer upstream of the Cas9-binding gRNA scaffold. Plasmids with successful gRNA insertion can be selected by blue-white screening of E. coli transformants. Download FIG S4, TIF file, 1.5 MB. Copyright © 2020 Rajaram et al. 2020 Rajaram et al. This content is distributed under the terms of the Creative Commons Attribution 4.0 International license.

To generate a pCasG-LacZ plasmid with a guide RNA sequence that targets PfAUBL, pCasG-LacZ was digested with BsaI. PfAUBL-specific guide RNAs were synthesized as oligonucleotides (Table S1), annealed, and inserted into the digested plasmid using In‐Fusion.

### Parasite transfections.

P. falciparum NF54^attB^ parasites were transfected using a previously described method with minor modifications (53). Briefly, red blood cells (RBCs) (350 μl) were electroporated with 75 μg each of the pCasG^PfAUBL^ plasmid and either the linearized pMG74^PfAUBL^ or pKD^PfAUBL^ plasmids. The electroporated RBCs were mixed with 1.5 ml of a mixed-stage culture at ∼4% parasitemia. Transfected cultures were maintained in CMA with 0.5 μM anhydrotetracycline (aTC) (Cayman Chemical) for the first 48 h, after which they were cultured in selective medium containing 2.5 μg/ml blasticidin (Corning) and 1.5 μM DSM-1 (Calbiochem) along with aTC for 7 days. Cultures were then switched to CMA with aTC until the reemergence of parasites, upon which they were transferred to CMA containing blasticidin and aTC. Single clones of PfAUBL^Apt^ parasites were isolated by limiting dilution in a 96-well plate.

### Genotyping and aptamer PCR.

A 3-μl volume of parasite culture or 10 ng of plasmid was used in 25-μl PCRs with Phusion High-Fidelity DNA polymerase (New England BioLabs [NEB]). To confirm pMG74^PfAUBL^ insertion into the 3′ UTR of PfAUBL, the following primer pairs were used: AUBL 5′ F + pMG74 R for the 5′ integration locus, and TetR-seq + AUBL 3′ R for the 3′ integration locus. To confirm pKD^PfAUBL^ insertion into the 3′ UTR of PfAUBL, the following primer pairs were used: AUBL 5′ F + NewApt-5R for the 5′ integration locus, and TetR-seq + AUBL 3′ R for the 3′ integration locus. To determine if any wild-type parasites were still present in the transfected populations, the primer pair AUBL 5′ F + AUBL 3’R was used.

The original aptamer array in the pMG74 plasmid and PfAUBL^Apt^ parasites was amplified using the primer pair Apt-1F + Apt-10R. The redesigned aptamer array in the pKD plasmid and PfAUBL^NewApt^ parasites was amplified using the primer pair NewApt-1F + NewApt-10R. Primer sequences are listed in Table S1 and the expected PCR product sizes are included in Fig. S1B.

### Growth assays.

Parasites were washed with 10 ml CMA three times to remove aTC from the culture medium and then cultured in CMA alone or CMA with 0.5 μM aTC. They were seeded in a 96‐well plate (Corning) at a 0.5% starting parasitemia and 2% hematocrit in a total volume of 250 μl per well, in quadruplicate for each medium condition. The plates were incubated in chambers gassed with 94% N_2_, 3% O_2_, 3% CO_2_ at 37°C. A 10-μl volume of each parasite sample was collected every day for blood smear analysis or flow cytometry, and the culture medium was exchanged every other day for 8 days.

For growth curve determination by flow cytometry, samples collected on days 0 to 4 were diluted 1:10 in phosphate-buffered saline (PBS) and stored in a 96‐well plate at 4°C. On day 4, the parasites were stained with SYBR green by transferring 10 μl of the 1:10 dilutions to a 96‐well plate containing 100 μl of 1× SYBR green (Invitrogen) per well in PBS. The plates were incubated at room temperature under gentle shaking in the dark for 30 min. Postincubation, 150 μl of PBS was added to each well to dilute unbound SYBR green dye. The samples were analyzed with an Attune Nxt flow cytometer (Thermo Fisher Scientific), with a 50-μl acquisition volume and a running speed of 25 μl/min, with 10,000 total events collected. The process was repeated on day 8 for samples collected on days 5 to 8.

### Western blotting.

Parasites were washed with CMA three times to remove aTC from the culture medium. Samples were collected immediately post wash, and then every 24 h for the next 4 days. The collected samples were centrifuged at 500 × *g* for 5 min at room temperature (RT) and pellets were stored at –20°C until the time of protein extraction. To isolate parasites from RBCs, the pellets were thawed and resuspended in 0.15% saponin for 5 min at RT. Lysed RBCs were removed by washing three times with 1× PBS. Parasite pellets were resuspended in NuPAGE LDS sample buffer (Thermo Fisher) containing 2% β-mercaptoethanol and boiled for 5 min. Proteins were resolved by SDS-PAGE on 4 to 12% gradient gels and transferred to nitrocellulose membranes. Membranes were blocked in 5% milk and probed overnight at 4°C with 1:2,500 rat anti-HA MAb 3F10 (Roche). They were then incubated for an hour at RT with 1:5,000 anti-rat horseradish peroxidase (HRP)-conjugated antibody (GE Healthcare). Protein bands were detected on X-ray film using SuperSignal West Pico chemiluminescent substrate (Thermo Scientific), according to the manufacturer’s protocol. For loading controls, membranes were stripped of antibody with 200 mM glycine (pH 2.0) for 5 min and reprobed with 1:25,000 mouse anti-aldolase mAB 2E11. After incubation with 1:10,000 anti-mouse HRP-conjugated antibody (GE Healthcare), proteins were detected as described above.

### Immunofluorescence assay.

Parasites were prepared for immunofluorescence assay (IFA) as previously described (54). Briefly, parasites were resuspended in a 1:1 fixative of 4% paraformaldehyde and 0.0075% glutaraldehyde in PBS for 30 min. Cells were permeabilized for 10 min with 1% Triton X-100 and then reduced for 10 min with 0.1g/liter NaBH_4_ in PBS. Cells were blocked for 2 h in a solution of 3% (wt/vol) bovine serum albumin (BSA) in PBS and then incubated at 4°C overnight with anti-ACP rabbit antibodies (1:500) (55) and rat anti-hemagglutinin (HA) MAb 3F10 (1:500) (Roche). Cells were washed three times with PBS, and then incubated for 2 h in a PBS solution containing 3% BSA, goat anti-rabbit Alexa Fluor 594 (1:500), and donkey anti-rat Alexa Fluor 488 (1:300) (Life Technologies). Cells were washed three times with PBS and mounted on coverslips with ProLong Gold DAPI antifade reagent (Life Technologies) and sealed with nail polish. A series of images spanning 5 μm were acquired with 0.2-μm spacing using a Zeiss AxioImager M2 microscope. The images were deconvolved with VOLOCITY software (PerkinElmer) to report a single image in the *z*-plane.

## References

[1] World Health Organization. 2019. World malaria report 2019. World Health Organization, Geneva, Switzerland. https://www.who.int/publications/i/item/world-malaria-report-2019.

[2] Böhme U, Otto T, Sanders M, Newbold C, Berriman M. 2019. Progression of the canonical reference malaria parasite genome from 2002–2019 [version 2; peer review: 3 approved]. Wellcome Open Res 4:58. doi:10.12688/wellcomeopenres.15194.2. 31080894 PMC6484455

[3] Ke H, Sigala PA, Miura K, Morrisey JM, Mather MW, Crowley JR, Henderson JP, Goldberg DE, Long CA, Vaidya AB. 2014. The heme biosynthesis pathway is essential for *Plasmodium falciparum* development in mosquito stage but not in blood stages. J Biol Chem 289:34827–34837. doi:10.1074/jbc.M114.615831. 25352601 PMC4263882

[4] Shears MJ, Botté CY, McFadden GI. 2015. Fatty acid metabolism in the *Plasmodium* apicoplast: drugs, doubts and knockouts. Mol Biochem Parasitol 199:34–50. doi:10.1016/j.molbiopara.2015.03.004. 25841762

[5] Ke H, Ganesan SM, Dass S, Morrisey JM, Pou S, Nilsen A, Riscoe MK, Mather MW, Vaidya AB. 2019. Mitochondrial type II NADH dehydrogenase of *Plasmodium falciparum* (PfNDH2) is dispensable in the asexual blood stages. PLoS One 14:e0214023. doi:10.1371/journal.pone.0214023. 30964863 PMC6456166

[6] Jewett TJ, Sibley LD. 2003. Aldolase forms a bridge between cell surface adhesins and the actin cytoskeleton in apicomplexan parasites. Mol Cell 11:885–894. doi:10.1016/s1097-2765(03)00113-8. 12718875

[7] Buscaglia CA, Coppens I, Hol WGJ, Nussenzweig V. 2003. Sites of interaction between aldolase and thrombospondin-related anonymous protein in *Plasmodium*. Mol Biol Cell 14:4947–4957. doi:10.1091/mbc.e03-06-0355. 14595113 PMC284797

[8] Baum J, Richard D, Healer J, Rug M, Krnajski Z, Gilberger T-W, Green JL, Holder AA, Cowman AF. 2006. A conserved molecular motor drives cell invasion and gliding motility across malaria life cycle stages and other apicomplexan parasites. J Biol Chem 281:5197–5208. doi:10.1074/jbc.M509807200. 16321976

[9] Ghosh AK, Coppens I, Gårdsvoll H, Ploug M, Jacobs-Lorena M. 2011. *Plasmodium* ookinetes coopt mammalian plasminogen to invade the mosquito midgut. Proc Natl Acad Sci U S A 108:17153–17158. doi:10.1073/pnas.1103657108. 21949403 PMC3193258

[10] Vega-Rodríguez J, Ghosh AK, Kanzok SM, Dinglasan RR, Wang S, Bongio NJ, Kalume DE, Miura K, Long CA, Pandey A, Jacobs-Lorena M. 2014. Multiple pathways for *Plasmodium* ookinete invasion of the mosquito midgut. Proc Natl Acad Sci U S A 111:E492–E500. doi:10.1073/pnas.1315517111. 24474798 PMC3910608

[11] Agrawal S, Chung D-WD, Ponts N, van Dooren GG, Prudhomme J, Brooks CF, Rodrigues EM, Tan JC, Ferdig MT, Striepen B, Le Roch KG. 2013. An apicoplast localized ubiquitylation system is required for the import of nuclear-encoded plastid proteins. PLoS Pathog 9:e1003426. doi:10.1371/journal.ppat.1003426. 23785288 PMC3681736

[12] Yeh E, DeRisi JL. 2011. Chemical rescue of malaria parasites lacking an apicoplast defines organelle function in blood-stage *Plasmodium falciparum*. PLoS Biol 9:e1001138. doi:10.1371/journal.pbio.1001138. 21912516 PMC3166167

[13] Ganesan SM, Morrisey JM, Ke H, Painter HJ, Laroiya K, Phillips MA, Rathod PK, Mather MW, Vaidya AB. 2011. Yeast dihydroorotate dehydrogenase as a new selectable marker for *Plasmodium falciparum* transfection. Mol Biochem Parasitol 177:29–34. doi:10.1016/j.molbiopara.2011.01.004. 21251930 PMC3057331

[14] Storm J, Sethia S, Blackburn GJ, Chokkathukalam A, Watson DG, Breitling R, Coombs GH, Müller S. 2014. Phosphoenolpyruvate carboxylase identified as a key enzyme in erythrocytic *Plasmodium falciparum* carbon metabolism. PLoS Pathog 10:e1003876. doi:10.1371/journal.ppat.1003876. 24453970 PMC3894211

[15] Jhun H, Walters MS, Prigge ST. 2018. Using lipoamidase as a novel probe to interrogate the importance of lipoylation in *Plasmodium falciparum*. mBio 9:e01872-18. doi:10.1128/mBio.01872-18. 30459194 PMC6247088

[16] Collins CR, Das S, Wong EH, Andenmatten N, Stallmach R, Hackett F, Herman JP, Müller S, Meissner M, Blackman MJ. 2013. Robust inducible Cre recombinase activity in the human malaria parasite *Plasmodium falciparum* enables efficient gene deletion within a single asexual erythrocytic growth cycle. Mol Microbiol 88:687–701. doi:10.1111/mmi.12206. 23489321 PMC3708112

[17] Knuepfer E, Napiorkowska M, van Ooij C, Holder AA. 2017. Generating conditional gene knockouts in *Plasmodium*—a toolkit to produce stable DiCre recombinase-expressing parasite lines using CRISPR/Cas9. Sci Rep 7:3881–3881. doi:10.1038/s41598-017-03984-3. 28634346 PMC5478596

[18] Birnbaum J, Flemming S, Reichard N, Soares AB, Mesén-Ramírez P, Jonscher E, Bergmann B, Spielmann T. 2017. A genetic system to study *Plasmodium falciparum* protein function. Nat Methods 14:450–456. doi:10.1038/nmeth.4223. 28288121

[19] Armstrong CM, Goldberg DE. 2007. An FKBP destabilization domain modulates protein levels in *Plasmodium falciparum*. Nat Methods 4:1007–1009. doi:10.1038/nmeth1132. 17994030

[20] de Azevedo MF, Gilson PR, Gabriel HB, Simões RF, Angrisano F, Baum J, Crabb BS, Wunderlich G. 2012. Systematic analysis of FKBP inducible degradation domain tagging strategies for the human malaria parasite *Plasmodium falciparum*. PLoS One 7:e40981. doi:10.1371/journal.pone.0040981. 22815885 PMC3397994

[21] Muralidharan V, Oksman A, Iwamoto M, Wandless TJ, Goldberg DE. 2011. Asparagine repeat function in a *Plasmodium falciparum* protein assessed via a regulatable fluorescent affinity tag. Proc Natl Acad Sci U S A 108:4411–4416. doi:10.1073/pnas.1018449108. 21368162 PMC3060247

[22] Roberts AD, Nair SC, Guerra AJ, Prigge ST. 2019. Development of a conditional localization approach to control apicoplast protein trafficking in malaria parasites. Traffic 20:571–582. doi:10.1111/tra.12656. 31094037 PMC6663561

[23] Prommana P, Uthaipibull C, Wongsombat C, Kamchonwongpaisan S, Yuthavong Y, Knuepfer E, Holder AA, Shaw PJ. 2013. Inducible knockdown of *Plasmodium* gene expression using the glmS ribozyme. PLoS One 8:e73783. doi:10.1371/journal.pone.0073783. 24023691 PMC3758297

[24] Meissner M, Krejany E, Gilson PR, de Koning-Ward TF, Soldati D, Crabb BS. 2005. Tetracycline analogue-regulated transgene expression in *Plasmodium falciparum* blood stages using Toxoplasma gondii transactivators. Proc Natl Acad Sci U S A 102:2980–2985. doi:10.1073/pnas.0500112102. 15710888 PMC548799

[25] Pino P, Sebastian S, Kim EA, Bush E, Brochet M, Volkmann K, Kozlowski E, Llinas M, Billker O, Soldati-Favre D. 2012. A tetracycline-repressible transactivator system to study essential genes in malaria parasites. Cell Host Microbe 12:824–834. doi:10.1016/j.chom.2012.10.016. 23245327 PMC3712325

[26] Ganesan SM, Falla A, Goldfless SJ, Nasamu AS, Niles JC. 2016. Synthetic RNA-protein modules integrated with native translation mechanisms to control gene expression in malaria parasites. Nat Commun 7:10727–10727. doi:10.1038/ncomms10727. 26925876 PMC4773503

[27] Belmont BJ, Niles JC. 2010. Engineering a direct and inducible protein-RNA interaction to regulate RNA biology. ACS Chem Biol 5:851–861. doi:10.1021/cb100070j. 20545348

[28] Mair GR, Braks JAM, Garver LS, Wiegant JCAG, Hall N, Dirks RW, Khan SM, Dimopoulos G, Janse CJ, Waters AP. 2006. Regulation of sexual development of *Plasmodium* by translational repression. Science 313:667–669. doi:10.1126/science.1125129. 16888139 PMC1609190

[29] Amberg-Johnson K, Hari SB, Ganesan SM, Lorenzi HA, Sauer RT, Niles JC, Yeh E. 2017. Small molecule inhibition of apicomplexan FtsH1 disrupts plastid biogenesis in human pathogens. Elife 6:e29865. doi:10.7554/eLife.29865. 28826494 PMC5576918

[30] Nasamu AS, Glushakova S, Russo I, Vaupel B, Oksman A, Kim AS, Fremont DH, Tolia N, Beck JR, Meyers MJ, Niles JC, Zimmerberg J, Goldberg DE. 2017. Plasmepsins IX and X are essential and druggable mediators of malaria parasite egress and invasion. Science 358:518–522. doi:10.1126/science.aan1478. 29074774 PMC5928414

[31] Spillman NJ, Beck JR, Ganesan SM, Niles JC, Goldberg DE. 2017. The chaperonin TRiC forms an oligomeric complex in the malaria parasite cytosol. Cell Microbiol 19:e12719. doi:10.1111/cmi.12719. PMC5429184 28067475

[32] Boucher MJ, Ghosh S, Zhang L, Lal A, Jang SW, Ju A, Zhang S, Wang X, Ralph SA, Zou J, Elias JE, Yeh E. 2018. Integrative proteomics and bioinformatic prediction enable a high-confidence apicoplast proteome in malaria parasites. PLoS Biol 16:e2005895. doi:10.1371/journal.pbio.2005895. 30212465 PMC6155542

[33] Garten M, Nasamu AS, Niles JC, Zimmerberg J, Goldberg DE, Beck JR. 2018. EXP2 is a nutrient-permeable channel in the vacuolar membrane of *Plasmodium* and is essential for protein export via PTEX. Nat Microbiol 3:1090–1098. doi:10.1038/s41564-018-0222-7. 30150733 PMC6158082

[34] Ke H, Dass S, Morrisey JM, Mather MW, Vaidya AB. 2018. The mitochondrial ribosomal protein L13 is critical for the structural and functional integrity of the mitochondrion in *Plasmodium falciparum*. J Biol Chem 293:8128–8137. doi:10.1074/jbc.RA118.002552. 29626096 PMC5971461

[35] Walczak M, Ganesan SM, Niles JC, Yeh E. 2018. ATG8 Is essential specifically for an autophagy-independent function in apicoplast biogenesis in blood-stage malaria parasites. mBio 9:e02021-17. doi:10.1128/mBio.02021-17. 29295911 PMC5750400

[36] Florentin A, Stephens DR, Brooks CF, Baptista RP, Muralidharan V. 2020. Plastid biogenesis in malaria parasites requires the interactions and catalytic activity of the Clp proteolytic system. Proc Natl Acad Sci U S A 117:13719–13729. doi:10.1073/pnas.1919501117. 32482878 PMC7306762

[37] Istvan ES, Das S, Bhatnagar S, Beck JR, Owen E, Llinas M, Ganesan SM, Niles JC, Winzeler E, Vaidya AB, Goldberg DE. 2019. *Plasmodium* Niemann-Pick type C1-related protein is a druggable target required for parasite membrane homeostasis. Elife 8:e40529. doi:10.7554/eLife.40529. 30888318 PMC6424564

[38] Rudlaff RM, Kraemer S, Streva VA, Dvorin JD. 2019. An essential contractile ring protein controls cell division in *Plasmodium falciparum*. Nat Commun 10:2181–2181. doi:10.1038/s41467-019-10214-z. 31097714 PMC6522492

[39] Tang Y, Meister TR, Walczak M, Pulkoski-Gross MJ, Hari SB, Sauer RT, Amberg-Johnson K, Yeh E. 2019. A mutagenesis screen for essential plastid biogenesis genes in human malaria parasites. PLoS Biol 17:e3000136. doi:10.1371/journal.pbio.3000136. 30726238 PMC6380595

[40] Fierro MA, Asady B, Brooks CF, Cobb DW, Villegas A, Moreno SNJ, Muralidharan V. 2020. An endoplasmic reticulum CREC family protein regulates the egress proteolytic cascade in malaria parasites. mBio 11:e03078-19. doi:10.1128/mBio.03078-19. PMC7042697 32098818

[41] Gupta A, Bokhari AAB, Pillai AD, Crater AK, Gezelle J, Saggu G, Nasamu AS, Ganesan SM, Niles JC, Desai SA. 2020. Complex nutrient channel phenotypes despite Mendelian inheritance in a *Plasmodium falciparum* genetic cross. PLoS Pathog 16:e1008363. doi:10.1371/journal.ppat.1008363. 32069335 PMC7048409

[42] Ling L, Mulaka M, Munro J, Dass S, Mather MW, Riscoe MK, Llinás M, Zhou J, Ke H. 2020. Genetic ablation of the mitoribosome in the malaria parasite *Plasmodium falciparum* sensitizes it to antimalarials that target mitochondrial functions. J Biol Chem 295:7235–7248. doi:10.1074/jbc.RA120.012646. 32273345 PMC7247301

[43] Nessel T, Beck JM, Rayatpisheh S, Jami-Alahmadi Y, Wohlschlegel JA, Goldberg DE, Beck JR. 2020. EXP1 is required for organisation of EXP2 in the intraerythrocytic malaria parasite vacuole. Cell Microbiol 22:e13168. doi:10.1111/cmi.13168. 31990132 PMC7138706

[44] Polino AJ, Nasamu AS, Niles JC, Goldberg DE. 2020. Assessment of biological role and insight into druggability of the *Plasmodium falciparum* protease Plasmepsin V. ACS Infect Dis 6:738–746. doi:10.1021/acsinfecdis.9b00460. 32069391 PMC7155168

[45] Zhang M, Wang C, Otto TD, Oberstaller J, Liao X, Adapa SR, Udenze K, Bronner IF, Casandra D, Mayho M, Brown J, Li S, Swanson J, Rayner JC, Jiang RHY, Adams JH. 2018. Uncovering the essential genes of the human malaria parasite *Plasmodium falciparum* by saturation mutagenesis. Science 360:eaap7847. doi:10.1126/science.aap7847. 29724925 PMC6360947

[46] Spork S, Hiss JA, Mandel K, Sommer M, Kooij TWA, Chu T, Schneider G, Maier UG, Przyborski JM. 2009. An unusual ERAD-like complex is targeted to the apicoplast of *Plasmodium falciparum*. Eukaryot Cell 8:1134–1145. doi:10.1128/EC.00083-09. 19502583 PMC2725561

[47] Ramya TN, Mishra S, Karmodiya K, Surolia N, Surolia A. 2007. Inhibitors of nonhousekeeping functions of the apicoplast defy delayed death in *Plasmodium falciparum*. Antimicrob Agents Chemother 51:307–316. doi:10.1128/AAC.00808-06. 17060533 PMC1797641

[48] Agrawal S, van Dooren GG, Beatty WL, Striepen B. 2009. Genetic evidence that an endosymbiont-derived endoplasmic reticulum-associated protein degradation (ERAD) system functions in import of apicoplast proteins. J Biol Chem 284:33683–33691. doi:10.1074/jbc.M109.044024. 19808683 PMC2785210

[49] Fellows JD, Cipriano MJ, Agrawal S, Striepen B. 2017. A plastid protein that evolved from ubiquitin and is required for apicoplast protein import in *Toxoplasma gondii*. mBio 8:e00950-17. doi:10.1128/mBio.00950-17. 28655825 PMC5487736

[50] Adjalley SH, Johnston GL, Li T, Eastman RT, Ekland EH, Eappen AG, Richman A, Sim BKL, Lee MCS, Hoffman SL, Fidock DA. 2011. Quantitative assessment of *Plasmodium falciparum* sexual development reveals potent transmission-blocking activity by methylene blue. Proc Natl Acad Sci U S A 108:E1214–E1223. doi:10.1073/pnas.1112037108. 22042867 PMC3223476

[51] Ghorbal M, Gorman M, Macpherson CR, Martins RM, Scherf A, Lopez-Rubio J-J. 2014. Genome editing in the human malaria parasite *Plasmodium falciparum* using the CRISPR-Cas9 system. Nat Biotechnol 32:819–821. doi:10.1038/nbt.2925. 24880488

[52] Swift RP, Rajaram K, Liu HB, Dziedzic A, Jedlicka AE, Roberts AD, Matthews KA, Jhun H, Bumpus NN, Tewari SG, Wallqvist A, Prigge ST. 2020. A mevalonate bypass system facilitates elucidation of plastid biology in malaria parasites. PLoS Pathog 16:e1008316. doi:10.1371/journal.ppat.1008316. 32059044 PMC7046295

[53] Deitsch K, Driskill C, Wellems T. 2001. Transformation of malaria parasites by the spontaneous uptake and expression of DNA from human erythrocytes. Nucleic Acids Res 29:850–853. doi:10.1093/nar/29.3.850. 11160909 PMC30384

[54] Gallagher JR, Matthews KA, Prigge ST. 2011. *Plasmodium falciparum* apicoplast transit peptides are unstructured in vitro and during apicoplast import. Traffic 12:1124–1138. doi:10.1111/j.1600-0854.2011.01232.x. 21668595 PMC3629917

[55] Gallagher JR, Prigge ST. 2010. *Plasmodium falciparum* acyl carrier protein crystal structures in disulfide-linked and reduced states and their prevalence during blood stage growth. Proteins 78:575–588. doi:10.1002/prot.22582. 19768685 PMC2805782

