# Supplementary figures and images for: Redesigned TetR-Aptamer System To Control Gene Expression in Plasmodium falciparum
Source: mSphere. 2020 Aug 12;5(4):e00457-20. doi: 10.1128/mSphere.00457-20 (PMC7426165; doi:10.1128/mSphere.00457-20)

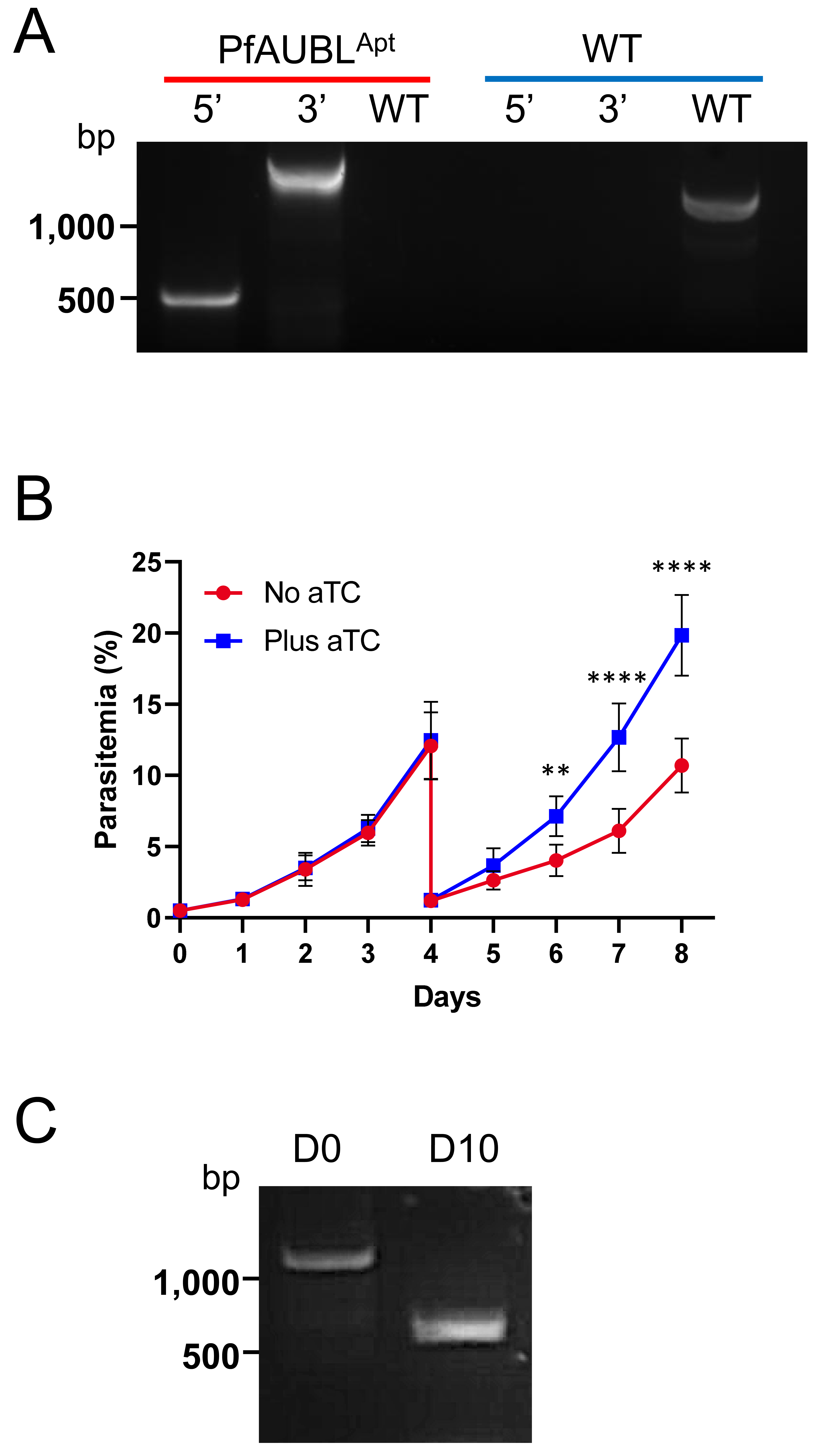

Supplement: FIG S2 [file mSphere.00457-20-sf002.tif]

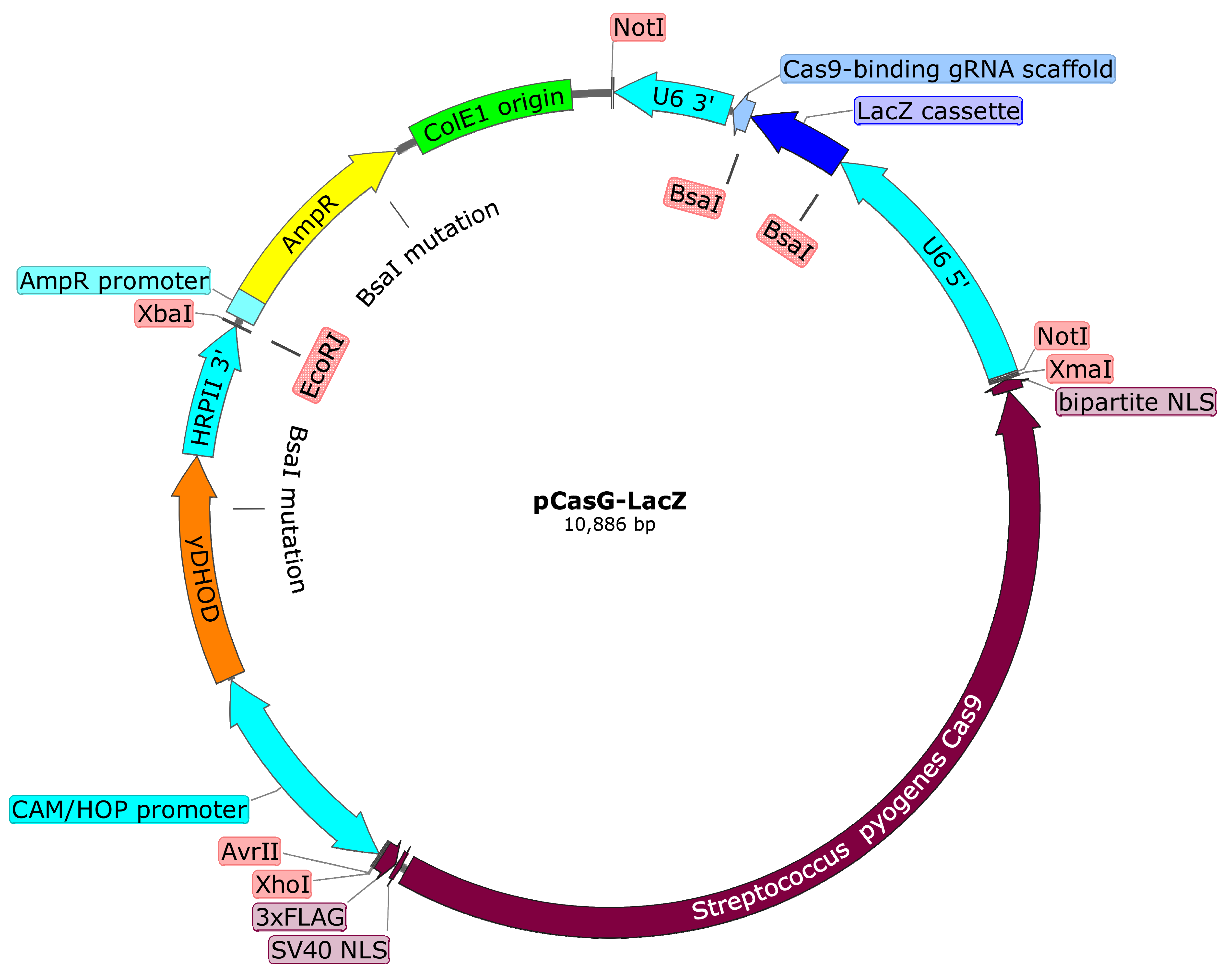

Supplement: FIG S4 [file mSphere.00457-20-sf004.tif]
