# Supplementary material for: Redesigned TetR-Aptamer System To Control Gene Expression in Plasmodium falciparum
Source: mSphere. 2020 Aug 12;5(4):e00457-20. doi: 10.1128/mSphere.00457-20 (PMC7426165; doi:10.1128/mSphere.00457-20)
Supplement: TABLE S1 [file mSphere.00457-20-st001.docx]

| **Primer Name** | **Sequence (5’ -> 3’)** |
| --- | --- |
| pCas.XhoBtg.F | 5P-TCGAGCCTAGGCCTGCTAGCACTGCGATGAC |
| pCas.XhoBtg.R | 5P-TTAAGTCATCGCAGTGCTAGCAGGCCTAGGC |
| pUF.NotI.F | 5P-AGCGGTACCGGTGCGGCCGCACGCGTC |
| pUF.NotI.R | 5P-CCGGGACGCGTGCGGCCGCACCGGTACC |
| BsaMut.GG.F | ACTAGGTCTCTCGTTTAAGACCTGAAATCAAAGTTATCGGTAC |
| BsaMut.GG.R | TAGTGGTCTCTACCGCGTGACCCACGCTCACCGGCTC |
| AUBL HA1.kd-F | GATATCGTCCACCTGGATATCGCAGACTATCCAAATCATATAAAGAAATTC |
| AUBL-HA1.kd-R | CATAAGGATAGACGTCTTGACACATCAAAATTTGTTGATAGG |
| AUBL HA2.kd-F | CCCTTTCCGGGCGCGCCGCTTTTATTATGCCTACACAAGGTTAG |
| AUBL-HA2.kd-R | GATATCCAGGTGGACGATATCCGTATTTTCTTATTTTCTTATTTTCATATTTTC |
| AUBL-gRNA.kd-F | TAAGTATATAATATTTGATGTGTCAATAAAATATCGTTTTAGAGCTAGAA |
| AUBL-gRNA.kd-R | TTCTAGCTCTAAAACGATATTTTATTGACACATCAAATATTATATACTTA |
| AUBL 5’ F | CTACGATTAGAAGTAGATTATCCTTTAATG |
| pMG74 R | GCATAATCAGGTACGTCATAAGGATAGACGTC |
| AUBL 3’ R | ATATTTATACGATAATATAAATGTAAGCGTC |
| Apt-1F | CTTATGACGTACCTGATTATGCAC |
| Apt-10R | GTAGACCCCATTGTGAGTACATAAATATATTATATAAACTAGACTAGG |
| NewApt-1F | GATGTACCAGATTACGCATAAGGG |
| NewApt-5R | CTCGCTATCAAGGAATCGAGTCC |
| NewApt-10R | CTAGACTAGGTTCCAAGATCTCCC |
| TetR-seq | GGCAGAAGTGAGTATGGTGCCTATC |

5P = 5’-phosphorylation.
