# Supplementary material for: Redesigned TetR-Aptamer System To Control Gene Expression in Plasmodium falciparum
Source: mSphere. 2020 Aug 12;5(4):e00457-20. doi: 10.1128/mSphere.00457-20 (PMC7426165; doi:10.1128/mSphere.00457-20)
Supplement: TEXT S1 [file mSphere.00457-20-s0001.docx]

pKD-PfAUBL plasmid sequence

agcttcgtacgctgcaggtcgagatttccccgaaaagtgccacctgggtccttttcatcacgtgggacattgtttaaacgagcagggacgtgcgcagctcaggggcatgatgtgactgtcgcccgtacatttagcccatacatccccatgtataatcatttgcatccatacattttgatggccgcacggcgcgaagcaaaaattacggctcctcgctgcagacctgcgagcagggaaacgctcccctcacagacgcgttgaattgtccccacgccgcgcccctgtagagaaatataaaaggttaggatttgccactgaggttcttctttcatatacttccttttaaaatcttgctaggatacagttctcacatcacatccgaacataaacaaccatgggtaaggaaaagactcacgtttcgaggccgcgattaaattccaacatggatgctgatttatatgggtataaatgggctcgcgataatgtcgggcaatcaggtgcgacaatctatcgattgtatgggaagcccgatgcgccagagttgtttctgaaacatggcaaaggtagcgttgccaatgatgttacagatgagatggtcagactaaactggctgacggaatttatgcctcttccgaccatcaagcattttatccgtactcctgatgatgcatggttactcaccactgcgatccccggcaaaacagcattccaggtattagaagaatatcctgattcaggtgaaaatattgttgatgcgctggcagtgttcctgcgccggttgcattcgattcctgtttgtaattgtccttttaacagcgatcgcgtatttcgtctcgctcaggcgcaatcacgaatgaataacggtttggttgatgcgagtgattttgatgacgagcgtaatggctggcctgttgaacaagtctggaaagaaatgcataagcttttgccattctcaccggattcagtcgtcactcatggtgatttctcacttgataaccttatttttgacgaggggaaattaataggttgtattgatgttggacgagtcggaatcgcagaccgataccaggatcttgccatcctatggaactgcctcggtgagttttctccttcattacagaaacggctttttcaaaaatatggtattgataatcctgatatgaataaattgcagtttcatttgatgctcgatgagtttttctaatcagtactgacaataaaaagattcttgttttcaagaacttgtcatttgtatagtttttttatattgtagttgttctattttaatcaaatgttagcgtgatttatattttttttcgcctcgacatcatctgcccagatgcgaagttaagtgcgcagaaagtaatatcatgcgtcaatcgtatgtgaatgctggtcgctatactgctgtcgattcgatactaacgccgccatccagtgtcgaaaacgagctcggtacctctagatgtaataaatatgttcttatatataatgagaaataaatatttaacatatgtttaaaaagaaaaatttaagatttacatgattaggaataaaaatattaaaagctatatattaatgttgtactttatgttcatattgtaaaagataaatataatttttattaaaatttatattattattattataagttcttagaaaatacattttgttttaaaataatactataaaaagaacaataataaaaataaaataaatacaattaaaacataattaaaaaagaaaatacagacagtaaaaaaaatcgctatcccataaattacaaaacatgaattaataaacatatattgtttattaataaaattgtaagttatttttatatatacataataaattaaagatactgaaacaataattttaaatgtaaataaatgaataatgatatgttttatatgattcattattctatatttataaggaagattacaaaaaaaaaattcatatgtattttttttgtaatttctgtgtttatgtttttatttttataatatttttaatctattattaaataaatttaatggaccggtttagccctcccacacataaccagagggcagcaattcacgaatcccaactgccgtcggctgtccatcactgtccttcactatggctttgatcccaggatgcagatcgagaagcacctgtcggcaccgtccgcaggggctcaagatgcccctgttctcatttccgatcgcgacgatacaagtcaggttgccagctgccgcagcagcagcagtgcccagcaccacgagttctgcacaaggtcccccagtaaaatgatatacattgacaccagtgaagatgcggccgtcgctagagagagctgcgctggcgacgctgtagtcttcagagatggggatgctgttgattgtagccgttgctctttcaatgagggtggattcttcttgagacaaaggcttggcgccggctggtcctggattttcttctacatctccacatgttaataaacttcctcttccttctccacttccctttgatatTcttctgaacacatgataatacaagtcccaggcttgaagtaagtccttaacatttcctgatttcatgtacttcctgcaccactcttgggcctccattaaatctcgaccataggcTtgattaaatgatgtttcctttaaagtttgaggtcccctttccatcatagcatgTaatggctctaacacctcaaacatacctttcacgttcctttctccaaagtacaaacgagatgcctcttcaagaccttcatgccacatctcatgccacaatattctTgaagccatactagctctacttaatattgctcctgctcctgctcctgctccactatttgcacttcttgttggatcacgcgtaacttttctttttttttttggtgccaTtggacctggattttcttcaacatcaccacatgttaataatgatcctctaccttcaccactaccgctagcggtatataaggatgggtcaatttcgtttggtattggttgtatttccgttcctagttctacttctattttatataaattaaaacgatcttcaaaagttataagattaatagctagtcctagatgtccgtatcttcctgatcttcctattctatgtaaataagtttcagaattctttgggaaatcaaaattgataacaacattgactgactgtatgtcgatacctcttgtgaatagatctgatgaaactaaacatctgcatgctccatttctaaaatcatgaaaaacacgattacgatgtgtttgtgacattcttgcatgaatgtaaaaagagctatatcctagttcggtaatttttttggctagtagttctaccctagtaatactattacagaagatgatagcttgattaatttgaagtttagcaaataatgtatttaaacaatgtactttttgtctttctttaacaaaagcataatattgtgttattccttttaaggttaattcatccataagatttatttcatgggcatctgataaataaatagctcgaaattcttttacagtcacaggaaaggtagcagaatacataagtatctgcttttcttttggtaaaaatttcattagttcttctactataggttgaaattcaggtgataataatttatctgcttcatccataaccataatatgacaacctgataaatttgctacatccttatttgctaagtctaatattcttcctggagtaccacataaaatatgaactacattatacaacctcattatatcttctcttaatgatgtaccaccggttgttaccatacactgtactttcatgtgttttcctaattccttaatcatagcagaggtctgtagggcaagttctcgcgtgggtaccaaaatgagtccttgaataaaatttttgtgggtattacatttctctagtaagggtatagcaaaagctgctgttttgcctgtaccattttttgcccttgctaaaatattttttccagccaaagctacaggtatactttcctcttgtataggtgatggtttctcatatcctttttcaaagatacccattaataattctctcttcaaaaaataatcttcaaattcattgcctttcgttttcgttacatcttctgtcttatatcttagatcttttaatggttccagaattttctttttccattcttcatctaatattatattatcatctattttgttataatttgaagaactattcaaagtatttgtattagcattagagttcgtacaattggttttataactactagtagacccactttcacatttaagttgtttttctaatccgcatatgatcaattcaaggccgaataagaaggctggctctgcaccttggtgatcaaataattcgatagcttgtcgtaataatggcggcatactatcagtagtaggtgtttccctttcttctttagcgacttgatgctcttgatcttccaatacgcaacctaaggtaaaatgccccacagcgctgagtgcatataacgcgttctctagtgaaaaaccttgttggcataaaaaggctaattgattttcgagagtttcatactgtttttctgtaggccgtgtacctaaatgtacttttgctccatcgcgatgacttagtaaagcacatctaaaacttttagcgttattacgtaaaaaatcttgccagctttccccttctaaagggcagaagtgagtatggtgcctatctaacatctcaatggctaaggcgtcgagcaaagcccgcttattttttacatgccaatacaatgtaggctgctctacaccaagcttctgggcgagtttacgggttgttaaaccttcgattccgacctcattaagcagctctaatgcgctgttaatcactttacttttatctaatctagacatcctaggtttattcgaaatgtgggaagaaaaaaaatataataaaaaaggaaaatttctttttaaagtaaaatatttatgtaaatatttatatattttattatatatatattattatatatatatatatatatatatataccaataaaataatataaaaatttttatgtatatctaaaaatatgtttttattaaaaggtaaaaataaacttattttttctttttttttaaaaaatatatatatatatatatatatatatataatattatatgagatatatattaaatattatattatttatataaatataatttttttcttaatttctcacgttgttaaaatttatatatttatatatgatatatattttttaatactttttaattttttttattatttttggtaattatcttattttattttaattttattattttatattaaatgatgctttttattttatattattattttttatttaaaatttttgctaaacatgaaattattttattatattttgtaaatacaaaataaaataatatataaatcaaaaaaaaaaaaaaagaaagaaagaaagaaagaaagaaagaaacaaagatgatatttaaaaaaaaaaagatattataaaaatataatattatatataaaatattatttttatataaatatatttatatatttatatatttatatatatgtatatatatatatatatatatataggtatagtatttattattttgttatatttatataatactcctttttgttattttttttttttttttctttaaattcatgcaaaaatttactataatattattatatttaactatatactatggaatactaaatatatatccaatggcccctttccgggcgcgccGCTTTTATTATGCCTACACAAGGTTAGTTATATATATATATATATATGTATGTATTTTATTTTTATTTTTCCTTATTTCAAGAAAGGAGAAATATAAATTAAATTAGATCTTTTTTACTTATTATAAGGATATATTATCTATTCATTTATTCATATATATATTCATTTATTTATTTATTTATTTATTTATTTTTTTTTTTGTTTAAAATAAAAAGCCTTATAGTTTCTTAAATATTTTTGTAAAATAAATATACAACAGTTGTTAATTTTTTTTTTTTTTTTTTTTTtAAGTAACATATAATTTaTTAATAAAATATAAAAATTAAAAAGTATGAAAATATGAAAATATGAAAATAAGAAAATAAGAAAATACGGATATCgtccacctgGATATCGCAGACTATCCAAATCATATAAAGAAATTCTTTTTATATTTATCTATTTTATTTCTAATATCTCCATTTAAACCTTTTTATAAATTTTCACACTTTCTCTTTTTTTCTGTACCAAATAATATACTTTTTTCAGGTATCATTCAAAGGGAAAAAAAATAATAATAAATAAATAAATAAATAAATAAATAAATAAATAAATATATATATATATATATATATATATATATATATATATATATATATATGTTAAATAATTAATATATACGTTTATATAATTTCCTTCTATTTCATATATTTTATTCGTAGGCTTTACAAATATTTTGTCTGCATCCTATCAACAAATTTTGATGTGTCAAgacgtcTCCGGATaCccttatgacgtacctgattatgcaggtTatccatacgatgttccagactatgctggaTacccttatgatgtaccagattacgcataagggcccactgtCtggcaaggcagagaaaggtcgatacggacggaatgtgatggccttgccagtacgatgtactgtccagaggcagagaaaggtcgatacggacggaatgtgatggcctctggacaacctgtaagacgtaggaggcagagaaaggtcgatacggacggaatgtgatggcctcctacgcaagatagcatacgctgaggcagagaaaggtcgatacggacggaatgtgatggcctcagcgtttcatagagtcgagtccaggcagagaaaggtcgatacggacggaatgtgatggcctggactcGATTCCTTGATAgcgagaggcagagaaaggtcgatacggacggaatgtgatggcctctcgctactgaacttgatcctccaggcagagaaaggtcgatacggacggaatgtgatggcctggaggacttcatttgcacgacgaaggcagagaaaggtcgatacggacggaatgtgatggccttcgtcgtagctagaactcacgtgaggcagagaaaggtcgatacggacggaatgtgatggcctcacgtgTGTCTACTGaACagagcaggcagagaaaggtcgatacggacggaatgtgatggcctgctctgactgacccgggagatcttggaacctagtctagtttatataatatatttatgtactcacaatggggtctacaaaaaaaaaaaaataaagatatatatatatatatattatctttcattttatcatcaccccaatatacatatatatatttttatatttttatatatttatatttttcttttatttatccataaaagaaaattaaatataaatttataccttttatttgaattaccatttgtataaaatataaaaaaaaaaaaaaataataaaaagaagaaaaaacgaaaaaagtaatatttaaataataaagatcaatatacataataaaaaaaaaaaaaaaaaaaaaaattaaaatttaaaaaattaaacattgaaataatttcatatacacacatatacatacatacatatatatatatatatatatatatttatacatttatgcatataaaatatattaacaacttatgtgtattcatatatatttttttacatatgtattgaaagactttactgagacatgaaatatctaataaccaaacgtttataaattctaattatatgtaaagcataatatagcttttgtctaatttaattccttaaaacgtatgtattaaaaatatagtaagatggggaaattataattttttttttaaaggtttttctttgatacagtaaaaaaaaaaaataaaaaaaaataataataataaaaaaataaaaaaataaaaaaaaataaaataattataaagatatatataattcctcccaaatacaaatgattaagtttaaaaattaataaataaatcaaaaaaaaatttaataataattgtattattttaagtgtagttaattcatcaaatagcatgcctgcaggtcgacgccagggttttcccagtcacgacgttgtaaaacgacggccagtgaattgtaatacgactcactatagggcgaattctggtttgtctggtcaaccaccgcggtctcagtggtgtacggtacaaacccgaattctggtttgtctggtcaaccaccgcggtctcagtggtgtacggtacaaacccggaattcgagctcgggcggccgccaagcttgagtattctatagtgtcacctaaatagcttggcgtaatcatggtcatagctgtttcctgtgtgaaattgttatccgctcacaattccacacaacatacgagccggaagcataaagtgtaaagcctggggtgcctaatgagtgagctaactcacattaattgcgttgcgctcactgcccgctttccagtcgggaaacctgtcgtgccagctgcattaatgaatcggccaacgcgcggggagaggcggtttgcgtattgggcgctcttccgcttcctcgctcactgactcgctgcgctcggtcgttcggctgcggcgagcggtatcagctcactcaaaggcggtaatacggttatccacagaatcaggggataacgcaggaaagaacatgtgagcaaaaggccagcaaaaggccaggaaccgtaaaaaggccgcgttgctggcgtttttccataggctccgcccccctgacgagcatcacaaaaatcgacgctcaagtcagaggtggcgaaacccgacaggactataaagataccaggcgtttccccctggaagctccctcgtgcgctctcctgttccgaccctgccgcttaccggatacctgtccgcctttctcccttcgggaagcgtggcgctttctcatagctcacgctgtaggtatctcagttcggtgtaggtcgttcgctccaagctgggctgtgtgcacgaaccccccgttcagcccgaccgctgcgccttatccggtaactatcgtcttgagtccaacccggtaagacacgacttatcgccactggcagcagccactggtaacaggattagcagagcgaggtatgtaggcggtgctacagagttcttgaagtggtggcctaactacggctacactagaagaacagtatttggtatctgcgctctgctgaagccagttaccttcggaaaaagagttggtagctcttgatccggcaaacaaaccaccgctggtagcggtggtttttttgtttgcaagcagcagattacgcgcagaaaaaaaggatctcaagaagatcctttgatcttttctacggggtctgacgctcagtggaacgaaaactcacgttaagggattttggtcatgagattatcaaaaaggatcttcacctagatccttttaaattaaaaatgaagttttaaatcaatctaaagtatatatgagtaaacttggtctgacagttaccaatgcttaatcagtgaggc
